# Computer Literacy and Health Locus of Control as Determinants for Readiness and Acceptability of Telepractice in a Head and Neck Cancer Population

**DOI:** 10.5195/ijt.2016.6203

**Published:** 2016-12-15

**Authors:** BENA CARTMILL, LAURELIE R. WALL, ELIZABETH C. WARD, ANNE J. HILL, SANDRO V. PORCEDDU

**Affiliations:** 1 CENTRE FOR FUNCTIONING AND HEALTH RESEARCH, QUEENSLAND HEALTH, AUSTRALIA; 2 SCHOOL OF HEALTH AND REHABILITATION SCIENCES, THE UNIVERSITY OF QUEENSLAND, AUSTRALIA; 3 SPEECH PATHOLOGY DEPARTMENT, PRINCESS ALEXANDRA HOSPITAL, QUEENSLAND HEALTH, AUSTRALIA; 4 CENTRE FOR RESEARCH EXCELLENCE IN TELEHEALTH, THE UNIVERSITY OF QUEENSLAND, AUSTRALIA; 5 RADIATION ONCOLOGY DEPARTMENT, PRINCESS ALEXANDRA HOSPITAL, QUEENSLAND HEALTH, AUSTRALIA; 6 SCHOOL OF MEDICINE, THE UNIVERSITY OF QUEENSLAND, AUSTRALIA

**Keywords:** Computer literacy, Health locus of control, Telepractice, Head and neck cancer

## Abstract

Understanding end-user populations is required in designing telepractice applications. This study explored computer literacy and health locus of control in head/neck cancer (HNC) patients to inform suitability for telerehabilitation. Sixty individuals with oropharygneal cancer were recruited. Computer literacy was examined using a 10-question survey. The Multidimensional Health Locus of Control Scale Form C (MHLC-C) examined perceptions of health “control”. Participants were mostly middle-aged males, from high socioeconomic backgrounds. Only 10% were non-computer users. Of the computers users, 91% reported daily use, 66% used multiple devices and over 75% rated themselves as “confident” users. More than half were open to using technology for health-related activities. High internal scores (MHLC-C) signified a belief that own behaviour influenced health status. HNC patients have high computer literacy and an internal health locus of control, both are positive factors to support telepractice models of care. This may include asynchronous models requiring heightened capacity for self-management.

The use of technology as an alternate mode for the delivery of healthcare education, assessment and rehabilitation is well established (Finch and Hill, 2014; Lea, Lockwood & Ringash, 2005; Murray, Burns, Tai, Lai, & Nazareth, 2005; Winters, 2002). Early investigations of technology-based models for patient education (van den Brink, Moorman, De Boer, Pruyn, Verwoerd, Van Bemmel, 2005), treatment monitoring (Cnossen et al., 2012; Head et al., 2009; Wall et al., 2015;), as well as delivery of therapy during (Cnossen et al., 2014) and in the post-treatment phase (Burns et al. 2012) have demonstrated good feasibility, favourable patient outcomes and high consumer satisfaction.

However, for future telepractice applications to be designed optimally, deeper understanding of the end-user population and their needs and skills is imperative to ensure appropriate integration of technology to replace or supplement in-person service delivery (Brennan & Barker, 2008; Pramuka & van Roosmalen, 2009). Human factors such as age, education, technology experience, functional status, and preference and readiness for health services have all been noted to impact on the delivery and receipt of both e-Health and telerehabilitation services (Brennan & Barker, 2008; Lea et al., 2005). Technology usability and accessibility (including experience in connecting, installing, recording/transmitting data) have been particularly noted as key factors influencing user perceptions and ultimately successful uptake of telepractice (Brennan & Barker, 2008; Pramuka & van Roosmalen, 2009; Sharma et al., 2013).

Within the HNC population, the issue of computer/technology use has not been reported for over a decade. In 2005, a large survey of HNC patients at a major cancer care centre in Toronto revealed 48% of respondents did not use computers (Lea, Lockwood, & Ringash, 2005). Furthermore, 67% reported they were not likely to access e-Health information, citing unfamiliarity with computers and lack of access to computers to be main factors influencing this decision. Another study published at the same time from the United States reported slightly higher computer use, with 71.6% of participants reporting access to a computer and 77% reporting knowing how to use it (Kagan, Clarek & Happ, 2005).

In addition to issues related to use of technology, limitations to the development of the therapeutic relationship (ie. a lack of shared space, differences in visual/sensory feedback) may also impact on the effectiveness of telepractice models (Brennan & Barker, 2008; Pramuka & van Roosmalen, 2009). Some literature has noted that the use of telepractice without proper support can lead to an increased sense of alienation in therapy by some patients (Bauer, 2010; Meredith, Firmin, & McAllister, 2015). This may be particularly applicable when using systems that are asynchronous, where there is a disconnect in data recording and communication between patient and clinician, and therefore less real time support (Pramuka & van Roosmalen, 2009). It is acknowledged therefore that patients need to play a more active role in engaging and self-managing the technology, as well as increased self-motivation to complete their therapy via an asynchronous method, for such a model to be successful.

It has been theorised that patients’ health-related attitudes, specifically the degree of control they believe they have over their health state, will influence the behaviours they will undertake in relation to their health condition (Wallston et al., 1976). In 1994, data was reported on the “health locus of control” (HLC) of a cohort of 93 patients receiving chemotherapy for various types of cancer. This data revealed that the group reported higher ratings on “external” HLC domains – indicating beliefs that chance, luck, or other people influence their health, as opposed to health being a function of ones own behaviour (“internal” HLC). Within the HNC population, it is recognised that self-efficacy and motivation to engage in rehabilitation during cancer treatment may be challenging, due to a multiplicity of factors such as the stress associated with diagnosis, and debilitating treatment-induced side effects (Shinn et al., 2013; van der Molen, et al., 2011). Therefore, the extent to which HNC patients feel in control of their health and are motivated to act in ways to improve and enhance their health, are likely to influence how they engage with telepractice interventions – particularly those designed for asynchronous delivery.

Whilst these initial studies have been instrumental in shaping early opinions towards the suitability of the HNC population to technology-based services, it is important to reaffirm whether these findings hold true for current patients. The past decade has witnessed considerable growth in the access, uptake and dissemination of technology within the general population (Australian Bureau of Statistics, 2016; International Telecommunications Union, 2015; File, 2013; File & Ryan, 2014). Furthermore, there has been a shift in the demographic profile of the HNC population, due to an increasing numbers of patients presenting with disease mediated by the human papilloma virus (HPV). The traditional archetype of an older male with low socioeconomic status and education who may oppose the use of technology in their healthcare, has been replaced with increasing numbers of patients who are younger, professional (D’Souza et al., 2010; Gillison et al., 2008), and therefore may be more likely to use technology for health-related activities (Lea et al., 2005). Hence the aim of this study was to explore current computer literacy and patient-perceived health locus of control with a cohort of patients with HNC, as potential determinants of readiness and appropriateness for technology-assisted service delivery models now and in the future.

## METHODS

### PARTICIPANTS

Participants were recruited from the Metro South Radiation Oncology Service (MSROS) – a tertiary cancer referral centre in Brisbane, Australia. Participants in the current study represent a sub-group of a larger ongoing RCT investigating the use of telepractice for prophylactic swallowing therapy during (chemo)radiotherapy ([C]RT) for HNC. As such, all participants were required to meet the eligibility criteria for receiving prophylactic swallowing therapy at MSROS: adults diagnosed with oropharyngeal HNC and planned for non-surgical treatment of curative-intent (C)RT. Exclusionary criteria included: (1) severe cognitive deficits; (2) non-English speaking; or (3) significant vision, hearing or physical dexterity impairments. No prior computing or technology skills were required. Ethical approval was obtained from the Metro South Human Research Ethics Committee in Brisbane, Australia (HREC/13/QPAH/153). Written informed consent was obtained for all eligible patients at the time of recruitment.

Sixty, eligible, consecutive participants were recruited between January 2014 and January 2016, with demographics summarised in Table 1. Participants were typically male, aged in their late-50s. The majority had a high socioeconomic status as determined by geographical location of residence (Australian Bureau of Statistics, 2011). All received definitive radiotherapy for oropharyngeal HNC with the majority receiving concomitant chemotherapy agents. Most patients had p16 (HPV) positive markers for virally mediated disease and presented with locally advanced lesions.

### PROCEDURE

Eligible patients were identified by review of weekly clinic lists and approached sequentially at their first radiotherapy planning appointment. All participants completed two outcome measures: a) a computer literacy survey and b) a health locus of control measure within the two weeks prior to or in the first week of their radiotherapy treatment.

### COMPUTER LITERACY

A purpose-built questionnaire was developed for the study, and was adapted from previous research evaluating the computer use of individuals with neurological language deficits (Finch & Hill, 2014; Appendix A). The questionnaire consisted of three main sections. The first section contained a series of tick box questions detailing the nature of participants’ current computer use, including common tasks where they used a computer or related technology (15 items) and the frequency of which these tasks were completed (*Daily, Weekly, Fortnightly, Monthly, Rarely, Never*). The second section contained more specific questions (yes/no, multiple choice, open-ended) including: types of computer devices they had experience with (1 item), the level of assistance they required (1 item), their attitudes towards computer use for everyday purposes (5 items), whether they had any experience in using computers for health-related activities (HRAs) (3 items) and their attitudes towards the use of technology for HRAs (2 items). The final section included two five-point Likert scale ratings of participants’ confidence *(Very confident, Somewhat confident, Unsure, Somewhat not confident, Not at all confident)* towards using a computer or related technology for a) general purposes and b) HRAs. Following completion of the survey, information from 3 key questions was used by the research team to classify participants into binary categories. A “low level” of computer literacy was indicated by nil current computer use, or some computer use but requiring substantial assistance to access computing technology. A “sufficient level” of computer literacy was indicated by: frequent use of computers (minimum weekly use) +/− using multiple technological devices or requiring nil assistance with using computing technology. This criteria was determined by the study team as the minimum skills needed to access and use a simple asynchronous telepractice application.

### HEALTH LOCUS OF CONTROL

The Multidimensional Health Locus of Control Scale – Form C (MHLC-C) (Wallston, Stein, & Smith, 1994) is a general purpose, condition-specific locus of control scale validated with a range of morbidities including cancer populations. It was utilised to determine patients’ beliefs regarding the degree of control they believed they had over their health condition. The MHLC-C consists of 18 Likert Scale (6-point) items across three subscales: (1) Internal HLC, which indicates a belief that one’s own behaviour influences one’s health status; (2) Chance HLC, which is the belief that one’s health condition is a matter of fate, luck or chance; and (3) Powerful Others HLC (including 2 subscales – Doctors and Other (powerful) people), which is the belief that other people, such as doctors, nurses, family and friends have control over one’s health status (Wallston et al., 1994).

### DATA ANALYSIS

Demographic and computer usage data were analysed descriptively using frequencies and percentages. Open-ended survey questions were analysed for pertinent themes. The four scales of the MHLC-C were examined descriptively using means, medians and standard deviations. Mean comparisons (t-test) for HLC were made with a historical cohort of 93 heterogeneous cancer patients receiving chemotherapy, as described by Wallston, Stein and Smith (1994). For all comparisons, *p*<0.05 indicated statistical significance.

## RESULTS

### COMPUTER LITERACY

**Figure 1:**
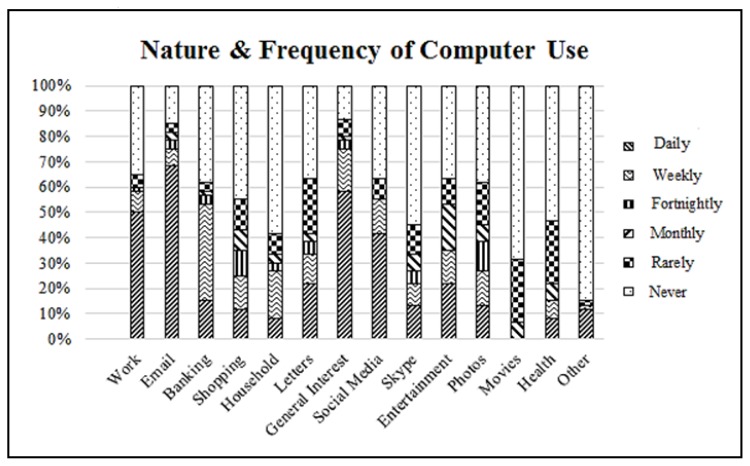
Frequency of computer-based tasks for everyday activities.

Overall, 90% (54/60) of participants reported using computers for general purposes, with 49/60 (82%) individuals reporting daily use (Table 2). The most common tasks were email, work and general interest/web surfing, with 50–68% of participants reporting daily use of computers for these tasks (Figure 1). Only 17% of participants reported that they required assistance to use computers. This assistance typically involved getting into/using computer programs and applications. Two thirds of respondents reported that they used multiple devices, with laptop computers, followed by tablets and smartphones the dominant devices (Table 2). Sub-analysis using the binary classification showed that 85% (n= 51) of patients demonstrated an overall “sufficient level” of computer literacy.

Participants’ attitudes towards computers and technology for general purposes were mostly positive. Respondents identified that computers offered a range of benefits, including: speed and convenience, the ability to access a wide range of information, ease of use, communication and interaction, and applications for work-related activities. A smaller number of participants also identified some challenges with using computers, particularly: technical difficulties and troubleshooting, safety and privacy, demands on time/tediousness, and lack of skills to use the technology effectively (Table 3).

With regard to the use of computers or related technology for health related activities, only 17% of participants reported that they had previous experience with computerised health applications (Table 2). This experience included completing health questionnaires, psychological/cognitive testing, weight and exercise tracking, and some therapy applications. Two participants also reported using computers for research into their condition and planned (chemo)radiotherapy treatment. All participants who reported using computers for health-related purposes stated that they liked doing so (n= 10). For those participants who didn’t have prior exposure to technology-enabled healthcare (n = 50), 25 individuals reported that they would be open to using technology for HRAs in the future. Overall, 78% (47/60) reported having confidence to use technology to manage their health.

Questions relating to participants’ confidence revealed 75% rated that they were confident using a computer or related technology for general purposes, with 23 participants reporting that they were very confident. Almost half (47%) of respondents reported that they were at least somewhat confident, with 12% rating themselves as very confident with using technology for HRAs.

Participants’ attitudes towards computers and technology for general purposes were mostly positive. Respondents identified that computers offered a range of benefits, including: speed and convenience, the ability to access a wide range of information, ease of use, communication and interaction, and applications for work-related activities. A smaller number of participants also identified some challenges with using computers, particularly: technical difficulties and troubleshooting, safety and privacy, demands on time/tediousness, and lack of skills to use the technology effectively (Table 3).

With regard to the use of computers or related technology for health related activities, only 17% of participants reported that they had previous experience with computerised health applications (Table 2). This experience included completing health questionnaires, psychological/cognitive testing, weight and exercise tracking, and some therapy applications. Two participants also reported using computers for research into their condition and planned (chemo)radiotherapy treatment. All participants who reported using computers for health-related purposes stated that they liked doing so (n= 10). For those participants who didn’t have prior exposure to technology-enabled healthcare (n = 50), 25 individuals reported that they would be open to using technology for HRAs in the future. Overall, 78% (47/60) reported having confidence to use technology to manage their health.

Questions relating to participants’ confidence revealed 75% rated that they were confident using a computer or related technology for general purposes, with 23 participants reporting that they were very confident. Almost half (47%) of respondents reported that they were at least somewhat confident, with 12% rating themselves as very confident with using technology for HRAs.

### HEALTH LOCUS OF CONTROL

Data from the MHLC-C are summarised in Table 4. Overall, participants reported highest scores on the Internal HLC domain and comparatively lower scores for the Chance and Powerful Others domains. The current cohort reported significantly higher internal scores than the comparison data presented by Wallston et al. (1994) (Table 4). Participants also demonstrated significantly lower external scores than the historical cohort for Chance domain and the Powerful Others-Doctors sub-domain. No significant difference was observed for the Powerful Others-Other People sub-domain.

## DISCUSSION

This study aimed to explore computer literacy and patient perceived health locus of control as potential determinants of suitability of the oropharyngeal HNC population to engage in telepractice and, whether this population consider the use of technology for HRAs as acceptable. Demographic data from the study cohort of oropharyngeal HNC were found to be younger, with higher SES, and commonly with presenting with HPV-mediated disease. Recent research has demonstrated that the incidence of patients with HPV-associated oropharyngeal cancers has increased by 225% over the last 30 years (Chaturvedi et al., 2011), and that the prevalence of oropharyngeal lesions positive for HPV biomarkers has been documented as 40–80% in the USA (Marur et al., 2010), and up to 90% in Europe (Nasman et al., 2009). Although speculatory, this shift toward a younger, higher SES HNC population may contribute to the recent change in the way this group engage with technology, approach health care and health services, compared to a decade ago.

Given their demographic profile, the demonstrated high levels of computer access and computer use in the surveyed cohort were not unexpected. Nearly all participants had computer access; most reported daily use of computers or related technology for general purposes and two thirds were using multiple devices. This reveals an overall higher level of technological competence than previously reported (Kagan et al, 2005; Lea et al. 2005). The discordance between the historical data and the current study most likely reflects both the continued dissemination and uptake of information technology by the global population over the last decade, and an intrinsic link between features of the HPV-positive demographic (younger age, higher SES) and computer literacy.

Although the current cohort were more active computer and technology users, the large majority of participants reported no prior exposure to technology in HRAs, though 43% reported that they would be willing to participate. Despite being a decade on, this aspect represented little change from the earlier studies. One possible explanation for this is that despite continued research into the applications of telepractice and e-Health, positive findings are not yet being successfully translated into routine clinical practice where patients can access such services. A systematic review by Or & Karsh (2009), which synthesised predictive factors of patient acceptance of health-related IT, demonstrated that consideration of patient-specific factors (e.g., prior exposure to technology) is important, however there is also a need to study the influence of environmental variables – such as organisational attitudes and support. Further research guided by theoretical frameworks which incorporate these factors may assist in improving our understanding of the acceptance and ultimate uptake of telepractice and e-Health services.

Participants in the current study demonstrated a high propensity for an internal HLC orientation. Several studies have shown that people with internal HLC are more likely to hold good health in higher importance (Wallhagen et al., 1994) and engage in behaviour that facilitates physical well-being (Wallston & Wallston, 1978). Internal HLC has also been associated with higher adherence to medical recommendations in the management of chronic diseases such as diabetes (Schlenk et al., 1984), and even survival time post-lung transplant (Burker et al., 2005). Results from the current cohort demonstrated significantly higher scores on the internal domain and significantly lower scores on external domains than the comparison cohort of cancer patients reported by Wallston et al. (1994). It is acknowledged that the comparisons that can be drawn between a homogenous population and a larger heterogenous sample are restricted. However, these findings submit that the current cohort of participants perceived that they had more central control of their health condition, a sentiment that may make them well-suited to care models which require a more active patient role in rehabilitation and greater ownership of their health status.

The high levels of technological competence and internal health locus of control exhibited by this study population demonstrate that patients with oropharyngeal HNC may exhibit suitability to telehealth/telepractice models which require a greater degree of therapeutic independence on behalf of the patient. The use of asynchronous telehealth, which uses store-and-forward technology to transmit data between patient and clinician without requiring their real-time presence in a rehabilitation session (Deshpande et al., 2009), may therefore be responsive to the intrinsic attributes of this cohort. Despite the awareness of the importance of early ongoing patient support for swallowing issues in the oropharyngeal HNC population, patients face numerous challenges accessing in person face-to-face speech pathology services due to staff/service constraints (Krisciunas, Sokoloff, Stepas, & Langmore, 2012; Lawson & Ward, 2014; Passfield, McQueen & Hulcombe, 2014; Roe et al., 2012). The potential for asynchronous telepractice to supplement clinical services by providing a supported, home-based model of care to HNC patients is therefore very promising, and may facilitate better patient access to evidence-based practices whilst minimising burden on clinical resources.

Limitations in the current study are acknowledged, namely the relatively small sample size, the unvalidated survey, and the homogeneous cohort of patients with oropharyngeal HNC. It is acknowledged that a proportion of patients will continue to present to cancer centres with the traditional demographic features accompanying HPV negative disease, such as older age, lower education and SES – factors which may affect their access to and engagement with technology. Additionally, although telepractice issues were examined primarily, it is recognised that multiple theoretical constructs and modifiable factors may have influenced readiness to adapt to telepractice, and were not explored in detail in the current study. Future research with larger sample sizes and exploring other HNC sites of disease which have lower documented prevalence of HPV involvement may allow more comprehensive modelling of patient factors which may predict appropriateness for telehealth interventions. This may assist in targeting populations for which technology-based health services may be the most suitable. This study also only assessed health locus of control immediately prior to the beginning of (C)RT treatment, therefore participants’ perceptions may change during the course of (C)RT. Future work exploring this issue longitudinally may glean poignant information regarding patients’ suitability to certain service-delivery models along the treatment continuum.

## CONCLUSION

This participant cohort with oropharyngeal HNC demonstrated high levels of computer literacy and an inherent suitability for therapy models that require active participation in their health and rehabilitation. The current suggest that patients with oropharyngeal HNC may be particularly responsive to technology-enabled models of care, technology-enabled healthcare and therapy applications both now and in the future.

## Figures and Tables

**Table 1:** Participant Demographics (n = 60)

Parameter		% (n)
Age		Mean = 57.78 Range = 20 – 73
Gender	Male	90 (54)
	Female	10 (6)
HPV Status	Positive	85 (51)
	Negative	12 (7)
	Unknown	3 (2)
Socioeconomic status (decile)*		Median = 7 Range = 1 – 10
Stage of Disease	I –II	2 (1)
	III – IV	98 (59)
Radiation Treatment	Conventional (70Gy/35#)	78 (40)
	Accelerated (DAHANCA protocol 68Gy/34#)	22 (13)
Concurrent chemotherapy	Yes	92 (55)
	No	8 (5)

* Australian Bureau of Statistics Socio-Economic Indexes for Areas – national population decile (Index of Relative Advantage and Disadvantage) based on geographical location of residence. 1 = most disadvantaged, 10 = most advantaged

**Table 2:** Computer Literacy Questionnaire (n = 60)

Parameter			% (n)
Current computer use		Yes	90 (54)
		No	10 (6)
Type of technology use		Desktop	40 (6)
		Laptop	80 (12)
		Tablet	47 (7)
		Smart phone	60 (9)
		>1 device	66 (10)
Prior exposure to technology for Health-related Activities		Yes	17 (10)
		No	83 (50)
Confidence with technology for:			
	General purposes	Very confident	38 (23)
		Somewhat confident	37 (22)
		Unsure	8 (5)
		Somewhat not confident	2 (1)
		Not at all confident	13 (8)
		No response	2 (1)
	Health-related Activities	Very confident	12 (7)
		Somewhat confident	35 (21)
		Unsure	22 (13)
		Somewhat not confident	7 (4)
		Not at all confident	18 (11)
		No response	7 (4)

**Table 3:** Perceived Benefits and Challenges from Perspective of Participants

Perceived Benefits		Perceived Disadvantages	

Themes	Examples	Themes	Examples
Practical uses	Social media Email Online shopping Work tool	Lack of skill	Not knowing new programs
Ease of use		Difficulty with troubleshooting	Malfunctions Wifi blackspots Crashing/freezing
Convenience	Saves time Portability	Security concerns	Hacking/viruses Unsavoury web content
Access to information	Endless knowledge Instant information	Time wasting	Sedentary activity Anti-social
Communication	Keep in touch with family and friends Immediacy of communication		

**Table 4:** Multidimensional Health Locus of Control – Form C Data Compared with Historical Cohort (Wallston et al., 1994)

		Current cohort n = 60		Validated Cancer Cohort n = 93	p
		Mean (SD)	Median (Range)	Mean (SD)	
Internal (6–36)		23.05 (6.03)	22.5 (10–36)	18.49 (5.72)	**<0.0001**
Chance (6–36)		15.13 (6.03)	14 (6–35)	19.81 (7.13)	**<0.0001**
Powerful Others (6–36)					
	Doctors (3–18)	15.00 (2.56)	15 (8–18)	15.91 (2.39)	**0.0268**
	Other People (3–18)	10.85 (3.33)	11 (3–18)	10.96 (3.96)	0.8588
